# Declines in Human Rhinovirus, Coronavirus, Parainfluenza, and Adenovirus Infections During the COVID‐19 Pandemic: Evidence From Household‐Based Cohort Studies in Lima, Peru

**DOI:** 10.1111/irv.70233

**Published:** 2026-02-05

**Authors:** Leigh M. Howard, Ana I. Gil, Lucie Ecker, Huiding Chen, Qingxia Chen, Rubelio Cornejo, Stefano Rios, Mayra Ochoa, Bia Peña, Omar Flores, Claudio F. Lanata, Carlos G. Grijalva

**Affiliations:** ^1^ Department of Pediatrics Vanderbilt University Medical Center Nashville Tennessee USA; ^2^ Instituto de Investigación Nutricional Lima Peru; ^3^ Department of Biostatistics Vanderbilt University Medical Center Nashville Tennessee USA; ^4^ Division of Pharmacoepidemiology, Department of Health Policy and Biomedical Informatics Vanderbilt University Medical Center Nashville Tennessee USA

## Abstract

In a household cohort enrolled during the COVID‐19 pandemic in Lima, Peru, detections of human rhinovirus, endemic coronaviruses, and parainfluenza viruses were significantly lower compared to these viral detections in a similar household cohort followed during the same seven epidemiological weeks in a period immediately preceding the COVID‐19 pandemic.

## 1 Background

Infections with certain viruses, such as respiratory syncytial virus (RSV) and influenza, were substantially reduced across many settings during the pandemic. Although some have proposed direct antagonistic interactions with SARS‐CoV‐2, these changes have been largely attributed to the implementation of nonpharmaceutical interventions (NPIs) and physical distancing measures to mitigate SARS‐CoV‐2 transmission [1, 2]. However, reported reductions in human rhinovirus (HRV) circulation were not as consistent or prolonged as reported for influenza or RSV [3, 4, 5, 6, 7]. Importantly, many of these observations were derived from population‐based studies or viral surveillance conducted in healthcare settings, while fewer studies report direct observations of community‐based detections of respiratory viruses [8, 9, 10]. We aimed to compare the detection of several common respiratory viruses in a household cohort enrolled during the COVID‐19 pandemic to detections from a household cohort enrolled in the same area during a period immediately preceding the COVID‐19 pandemic in Lima, Peru.

## 2 Methods

### 2.1 Study Design and Setting

We conducted two prospective household‐based cohort studies in San Juan de Lurigancho, a densely populated peri‐urban district in Lima, Peru. Enrollment and follow‐up of the first cohort took place from December 2019 until March 2020 (prepandemic), and the second cohort was enrolled and followed from November 2020 to February 2021 (pandemic) (22). Household members who provided informed consent were followed through twice‐weekly household visits to assess acute respiratory illness (ARI) symptoms and to obtain respiratory samples.

Peru was among the first countries to implement government‐imposed lockdowns in March 2020, including school closures, cancellations of public gatherings, and stay‐at‐home orders (23). Schools remained closed for 2 years, fully reopening in March 2022. Peru had one of the most strict and longest lockdown responses to the pandemic, compared to other countries where implementation of mitigation strategies was limited, delayed, or variably enforced (24). The pandemic cohort was followed during this period of strict lockdown.

### 2.2 Household Eligibility

Potentially eligible households were identified through a community‐wide census prior to the study. Households were excluded if they planned to move outside the area and would not be available for follow‐up. After written informed consent was obtained, along with written assent for participants 8 to 17 years of age, individuals were enrolled and follow‐up continued through the end of study, voluntary withdrawal from the study, loss to follow‐up, or death. For the prepandemic cohort, households were eligible if they contained at least one child 5 to 60 months of age and at least one other household member willing to enroll. For the pandemic cohort, households with at least one child (< 18 years), one younger adult (18–50 years), and one older adult (> 50 years) were eligible. Three members from each household of varying sizes were enrolled into each cohort.

From all enrolled households, a subset was selected for inclusion in this analysis as previously described [11]. As part of another study assessing the incidence of SARS‐CoV‐2, we first selected a convenience sample of households from the pandemic cohort for sequencing if a SARS‐CoV‐2 infection occurred in any family member. Corresponding households matched by household composition and calendar period of observation were then selected from the prepandemic cohort. Some households from the prepandemic cohort had occurrences of an ARI, but not all.

### 2.3 Data Collection

Trained field workers visited households twice weekly to assess for ARI symptoms each day of surveillance. A one‐time structured questionnaire was administered to assess basic demographics and medical history. No COVID‐19 vaccination occurred in our population during the study period.

### 2.4 Specimen Collection

Weekly nasopharyngeal (NP) swabs were collected from each enrolled household member by trained field workers, regardless of respiratory symptoms, using rayon swabs in skim milk–tryptone–glucose–glycerin (STGG) media, transported in cold boxes to a local laboratory where they were frozen at −80°C until selected samples were shipped on dry ice from Peru to Vanderbilt University Medical Center, then stored at −80°C.

### 2.5 Viral Detection

Specimens from individuals in selected households underwent identification of several respiratory viruses, including HRV species A, B, or C, human coronaviruses (HCoV), parainfluenza viruses 1–4 (HPIV), and adenovirus (AdV) using the Illumina Respiratory Pathogen ID/AMR Panel as previously described [11].

### 2.6 Statistical Analysis

We restricted the analytic population to observations from individuals < 75 years old followed during epidemiological Weeks 1–7 to ensure that observation time and age distribution were comparable between cohorts. We compared the crude prevalence of detections of each virus between the cohorts during overlapping epidemiological weeks (Weeks 1–7). We also plotted the timing of HRV detections in individuals clustered by household in the prepandemic cohort.

Given the infrequency of HRV detections in the pandemic cohort, we applied logistic regression with a Firth's penalized likelihood method to estimate the odds of HRV detection and its 95% confidence interval (CI) in the pandemic compared to the reference prepandemic group, adjusting for sex. For HCoV, HPIV, and AdV, since there were no observations for these viruses in the pandemic cohort, Fisher Exact test was applied with Haldane–Anscombe correction [12] to compare odds of virus detection between prepandemic and pandemic cohorts. To demonstrate the impact of age, we plotted the predicted probabilities of detecting each virus against age. Statistical tests were performed using R software version 4.3.3 with packages *logistf* and *ggplot*. Two‐sided *p*‐values of < 0.05 were considered statistically significant.

## 3 Results

Overall, 120 households (395 individuals) were enrolled in the prepandemic cohort, and 44 households (132 individuals) were enrolled in the pandemic cohort. For this study, we selected a subset of 16 households (63 individuals contributing 414 NP specimens) in the prepandemic cohort and 16 households (51 individuals contributing 416 NP specimens) from the pandemic cohort (Figure S1). After exclusion of observations from individuals aged > 75 years and/or outside epidemiological Weeks 1–7, 663 specimens remained for analysis (407 prepandemic cohort and 256 pandemic). The median ages of the prepandemic and pandemic cohorts were 13.3 years (interquartile range [IQR] 4.5–36.8) and 26.9 years (IQR 4.6–55.2; *p* < 0.01), respectively (Tables S1 and S2), and the proportion of females was higher in the pandemic cohort (80.5%) compared to the prepandemic cohort (67.8%, *p* < 0.01).

**FIGURE 1:**
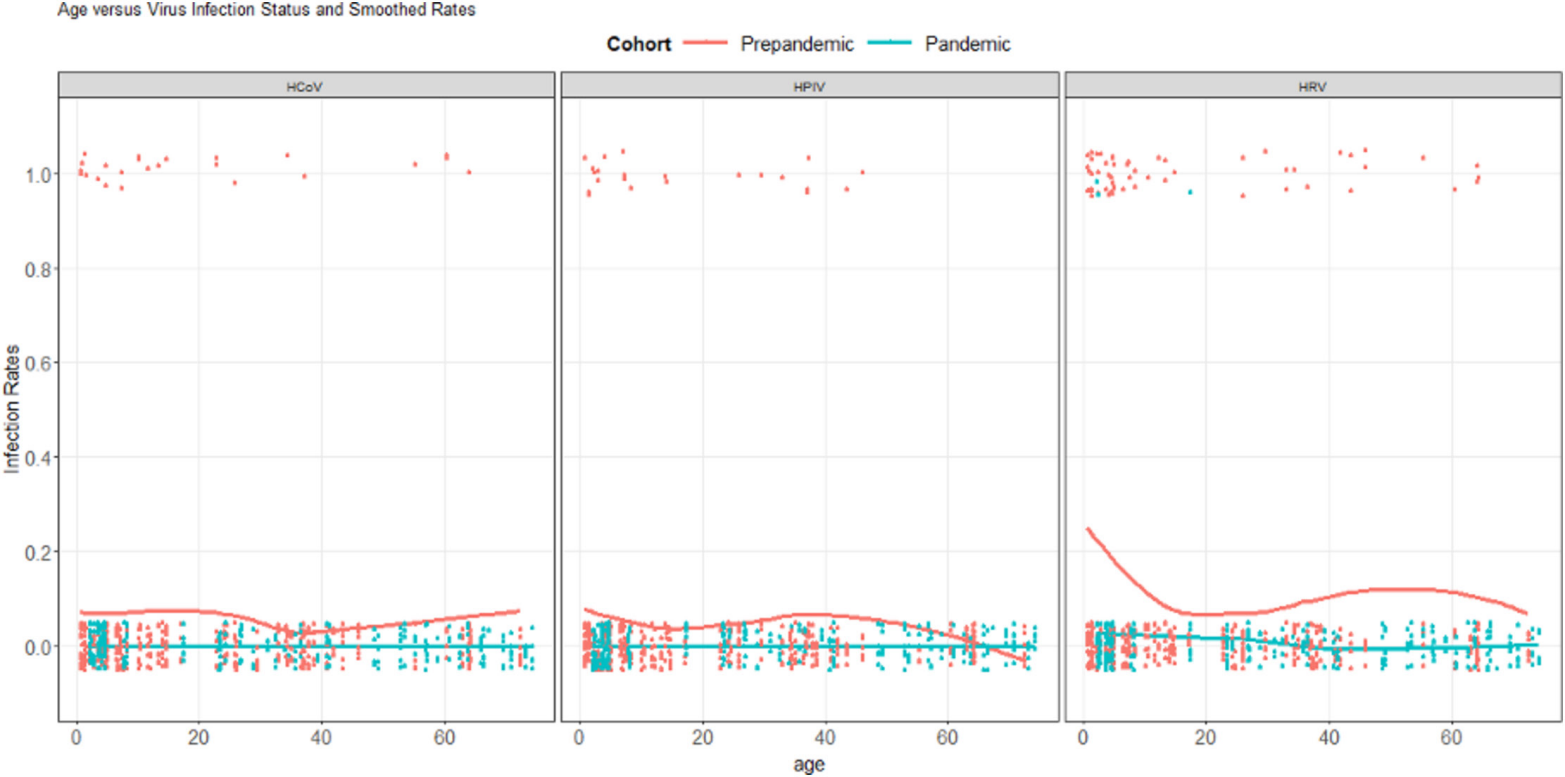
Estimated trend of detection of HRV, HCoV, and HPIV by age (years) in the prepandemic and pandemic cohorts in Lima, Peru (1.0: HRV detection; 0.0: negative HRV result).

HRV was detected in 57/407 (14.0%) samples from the prepandemic cohort and 3/256 (1.2%) of samples in the pandemic cohort. The odds of HRV detection during the pandemic were significantly lower than during the prepandemic period (adjusted odds ratio [aOR]: 0.10, 95% CI: 0.03–0.30). The prepandemic prevalence of detection of HCoV, HPIV, and AdV was 6.1% (25/407), 5.4% (22/407), and 1.5% (6/407), respectively, with no detections of these viruses in the 256 pandemic cohort specimens. The odds of HCoV and HPIV detection during the pandemic were significantly lower than during the prepandemic period (HCoV OR 0.06, *p* < 0.001, HPIV OR 0.07, *p* < 0.001). The odds of AdV detection did not significantly differ in the pandemic versus the prepandemic cohort (OR 0.22, *p* = 0.16). The predicted probabilities of virus detection by age for prepandemic and pandemic cohorts are displayed in Figure 1A–C.

Detections of HRV, clustered by household in the prepandemic period, are displayed in Figure S2, and HRV detections clustered by household in the pandemic period are displayed in Figure S3.

## 4 Discussion

Detections of HRV, HCoV, and HPIV were significantly reduced during a period of strict lockdown measures for the COVID‐19 pandemic among households in Lima, Peru. While declines in detections of many respiratory viruses during the early COVID‐19 pandemic were widely reported, some studies suggested that HRV circulation was only minimally disrupted early in the pandemic and returned to typical levels promptly after strict NPIs were lifted [3, 5]. These reports, combined with our finding that HRV activity (new household HRV introductions and secondary household HRV infections) was profoundly suppressed later in the first year of the pandemic, suggest that this reduction may be at least partially attributable to the more restrictive and prolonged implementation of NPIs in our study region. Others have suggested that the persistence of HRV during the pandemic in some settings may be attributable to unique properties of HRV (and potentially other nonenveloped viruses), including resistance to disinfection, the ability to survive on surfaces for prolonged periods, or interactions with other respiratory viruses [13].

Strengths of our study include observations from a community‐based household cohort with systematic collection of respiratory specimens without regard to symptoms. Our study was also subject to some limitations, including a small number of included households/participants, the relatively short interval of follow‐up for both cohorts, differences in the age distributions of the cohorts, although this was accounted for in our multivariable analysis, limited generalizability to settings with shorter or less strict NPI implementation, and limited ability to evaluate viruses with typical winter circulation since our study took place in the late‐spring/summer months.

In conclusion, detections of HRV, HCoV, and HPIV were significantly reduced during the COVID‐19 pandemic compared to a corresponding period the year before the pandemic. Future studies are needed to fill important gaps in our understanding of drivers of circulation of HRV and other respiratory viruses in order to prevent transmission of these viruses and their associated burden of illness.

## Author Contributions

**Leigh M. Howard:** conceptualization, investigation, data curation, methodology, writing‐original draft, writing‐review and editing, formal analysis, project administration, funding acquisition, supervision, visualization. **Ana I. Gil:** project administration, methodology, data curation, writing‐review and editing, investigation. **Lucie Ecker:** methodology, data curation, writing‐review and editing, investigation. **Huiding Chen:** methodology, formal analysis, visualization, data curation, writing‐review and editing, investigation. **Qingxia Chen:** methodology, formal analysis, visualization, data curation, writing‐review and editing, investigation. **Rubelio Cornejo:** methodology, data curation, writing‐review and editing, investigation. **Stefano Rios:** methodology, data curation, writing‐review and editing, investigation. **Mayra Ochoa:** methodology, data curation, writing‐review and editing, investigation. **Bia Peña:** methodology, data curation, writing‐review and editing, investigation. **Omar Flores:** methodology, data curation, writing‐review and editing, investigation. **Claudio F. Lanata:** conceptualization, project administration, methodology, data curation, writing‐review and editing, investigation. **Carlos G. Grijalva:** project administration, methodology, data curation, writing‐review and editing, investigation, supervision. All authors approve the final version to be published.

## Funding

This work was supported by the National Institute for Allergy and Infectious Diseases ([NIAID] Grant Numbers D43TW012468 [C.F.L. and C.G.G.], R21AI171901 [C.G.G. and L.M.H.] and K24AI148459 [C.G.G.]).

## Ethics Statement

This study was approved by the Institutional Review Board at Vanderbilt University Medical Center and the Instituto de Investigacion Nutricional ethics committee. Individuals enrolled voluntarily and received no compensation for their participation. Caregivers provided written informed consent for children.

## Conflicts of Interest

C.G.G. has received consulting fees from Merck and GSK. C.F.L. receives funding from HilleVax for work not related to the present study and is consultant to HilleVax and Merck.

## Supporting information


**Figure S1:** Cohort enrollment and household selection.
**Table S1:** Characteristics of prepandemic and pandemic cohorts, San Juan de Lurigancho, Lima, Peru.
**Table S2:** Characteristics of included and excluded swabs, San Juan de Lurigancho, Lima, Peru.
**Figure S2:**. Detection of HRV in individuals over time in the prepandemic cohort, with individual lines representing timelines of specimen collection for each individual, clustered by household. Lines color‐coded by age group. Gray diamonds indicate specimens that tested negative for HRV. Red diamonds and blue squares represent detections of HRV‐A and HRV‐C, respectively.
**Figure S3:**. Detection of HRV in individuals over time in the pandemic cohort, clustered by household. Lines color‐coded by age group. Gray diamonds indicate specimens that tested negative for HRV. Red diamonds represent detections of HRV (all HRV detections in the pandemic cohort were HRV‐A).

## Data Availability

The data that support the findings of this study are available on request from the corresponding author. The data are not publicly available due to privacy or ethical restrictions.
