# Supplementary material for: Declines in Human Rhinovirus, Coronavirus, Parainfluenza, and Adenovirus Infections During the COVID‐19 Pandemic: Evidence From Household‐Based Cohort Studies in Lima, Peru
Source: Influenza Other Respir Viruses. 2026 Feb 5;20(2):e70233. doi: 10.1111/irv.70233 (PMC12875687; doi:10.1111/irv.70233)

Supplementary Appendix

Supplementary Figure 1. Cohort enrollment and household selection

Previously reported in Charnogursky et al., Open Forum Infect Dis, 2025 Mar 20;12(4):ofaf157.

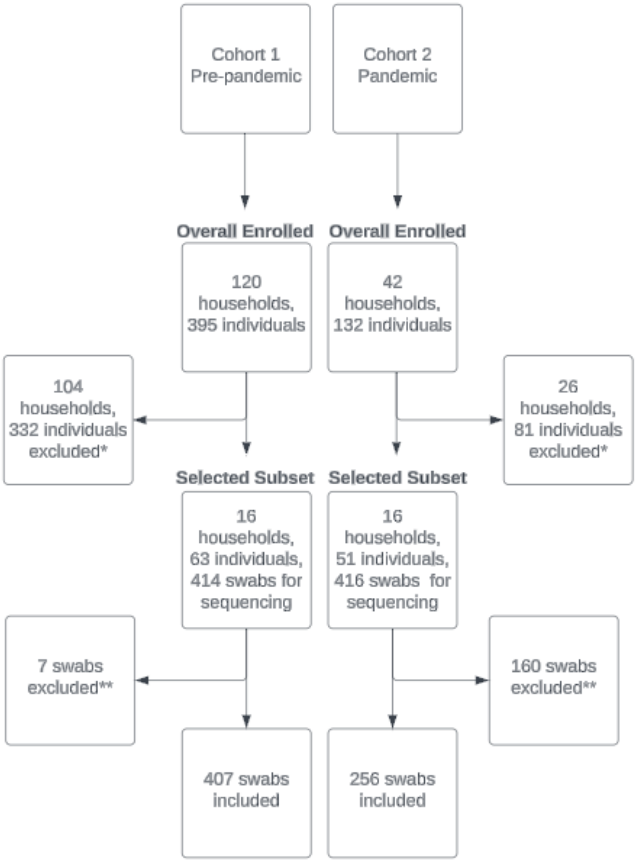

*Swabs included if met selection criteria of similar household composition or overlapping calendar weeks
**Swabs excluded if contributed during non-overlapping epidemiologic weeks and from individuals >75 years of age

Supplementary Table 1. Characteristics of pre-pandemic and pandemic cohorts, San Juan de Lurigancho, Lima, Peru

Previously reported in Charnogursky et al., Open Forum Infect Dis, 2025 Mar 20;12(4):ofaf157.

	Pre-pandemic cohort	Pandemic cohort	Test-statistic
**Number of participants**	63	51	
**Epidemiological week of specimen collection for included specimens**			$P < 0.01$
Median	4	5	
Range	1—7	1—7	
Mean±SD	3.94±1.97	4.58±1.75	
**Median household size** (interquartile range)	4 (2.42—5.17)	5 (4.0—6.58)	$P < 0.01$
Age in years, median (IQR) [Range]	13.3 (4.5—36.8) [0.7-72.4]	26.9 (4.6—55.2) [2.1-74.1]	$P < 0.01$
Age group, n(%)			
0-4 years	123/407 (30.22)	79/256 (30.86)	
5-17 years	109/407 (26.78)	20/256 (7.81)	
18-44 years	123/407 (30.22)	68/256 (26.56)	
45+ years	52/407 (12.78)	89/256 (34.77)	
Female sex, n(%)	276/407 (67.81)	206/256 (80.47)	$P < 0.01$
Number of NP swabs before exclusion	414	416	
Number of NP swabs after exclusion	407	256	

Supplementary Table 2. Characteristics of included and excluded swabs, San Juan de Lurigancho, Lima, Peru

	Swabs included (n=663)	Swabs excluded (n=167)
**Number of participants**	113	47
**Epidemiological week of specimen collection for included specimens**		
Median	4	9
Range	1—7	1-12, 52-53
Mean±SD	4.19±1.91	-
**Median household size** (interquartile range)	3 (3—4)	3 (3—3)
Age in years, median (IQR) [Range]	22.8 (4.6—40.9) [0.7-74.1]	35.7(4.6—57.0) [2.1-86.2]
Age group, n(%)		
0-4 years	202/663 (30.47)	51/167 (30.54)
5-17 years	129/663 (19.46)	5/167 (3.00)
18-44 years	191/663 (28.81)	44/167 (26.35)
45+ years	141/663 (21.27)	67/167 (40.12)
Female sex, n(%)	482/663 (72.70)	143/167 (80.47)

Supplementary Figure 2. Detection of HRV in individuals over time in the pre-pandemic cohort, with individual lines representing timelines of specimen collection for each individual, clustered by household. Lines color-coded by age group. Gray diamonds indicate specimens that tested negative for HRV. Red diamonds and blue squares represent detections of HRV-A and HRV-C, respectively.

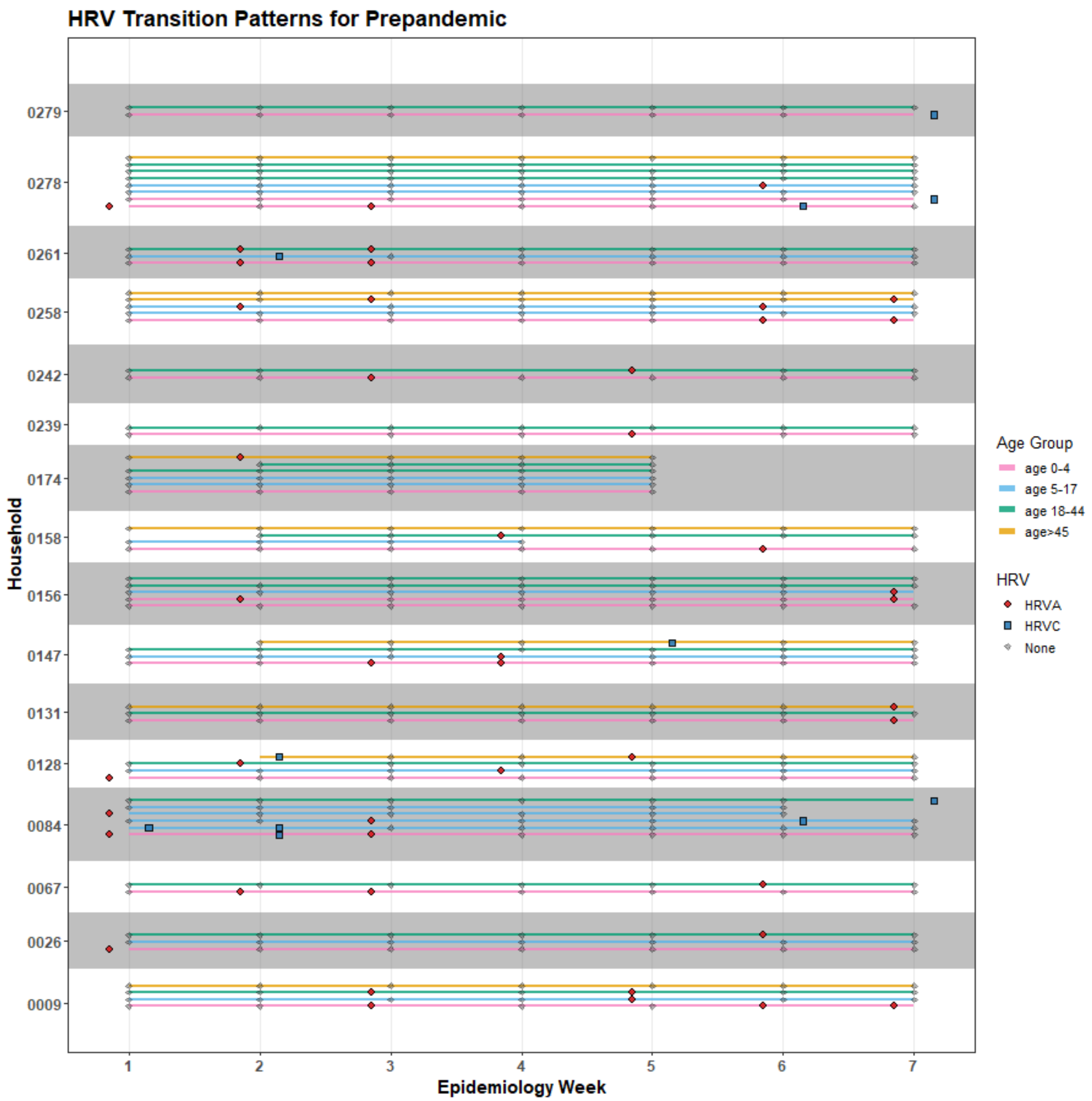

Supplementary Figure 3. Detection of HRV in individuals over time in the pandemic cohort, clustered by household. Lines color-coded by age group. Gray diamonds indicate specimens that tested negative for HRV. Red diamonds represent detections of HRV (all HRV detections in the pandemic cohort were HRV-A).

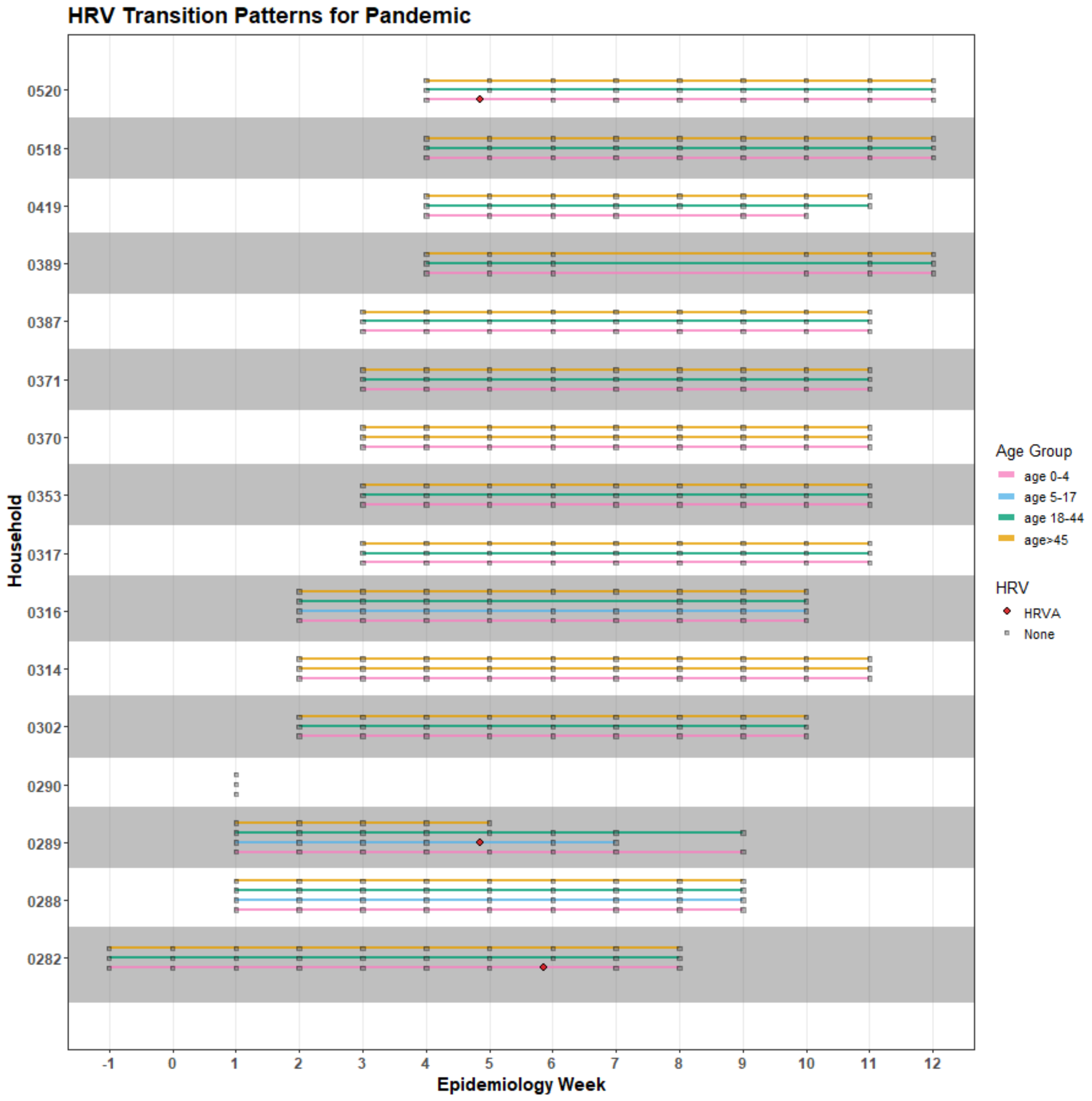

Supplement: Supplementary file 1 — Figure S1: Cohort enrollment and household selection. Table S1: Characteristics of prepandemic and pandemic cohorts, San Juan de Lurigancho, Lima, Peru. Table S2: Characteristics of included and excluded swabs, San Juan de Lurigancho, Lima, Peru. Figure S2:. Detection of HRV in individuals over time in the prepandemic cohort, with individual lines representing timelines of specimen collection for each individual, clustered by household. Lines color‐coded by age group. Gray diamonds indicate specimens that tested negative for HRV. Red diamonds and blue squares represent detections of HRV‐A and HRV‐C, respectively. Figure S3:. Detection of HRV in individuals over time in the pandemic cohort, clustered by household. Lines color‐coded by age group. Gray diamonds indicate specimens that tested negative for HRV. Red diamonds represent detections of HRV (all HRV detections in the pandemic cohort were HRV‐A). [file IRV-20-e70233-s001.pdf]
